# Effects of Tartary Buckwheat Bran Flour on Dough Properties and Quality of Steamed Bread

**DOI:** 10.3390/foods10092052

**Published:** 2021-08-31

**Authors:** Sheng Zhang, Si Chen, Sheng Geng, Changzhong Liu, Hanjun Ma, Benguo Liu

**Affiliations:** Henan Institute of Science and Technology, School of Food Science, Xinxiang 453003, China; 18738591585@163.com (S.Z.); chensi0856@126.com (S.C.); gengshenggs@126.com (S.G.); liuchangzhong68@163.com (C.L.); xxhjma@126.com (H.M.)

**Keywords:** Tartary buckwheat, bran, antioxidant activity, rheological properties, steamed bread

## Abstract

Steamed bread is a traditional staple food of China. Replacing wheat flour (WF) with Tartary buckwheat is expected to improve the nutritional value of steamed bread. In this study, Tartary buckwheat flour (TBF), Tartary buckwheat bran flour (TBBF), and Tartary buckwheat core flour (TBCF) were prepared, their composition and physicochemical properties were compared. It was found that TBBF had higher protein and rutin contents, so its antioxidant activity and dough rheological properties were obviously superior to those of TBF and TBCF. When TBBF was mixed with WF, its weight proportion in the blend (W_bran_) had a significant effect on the dough rheological properties. When W_bran_ was 30%, the dough had the optimal mixing tolerance, and when W_bran_ exceeded 30%, it caused dilution effect, weakening the gluten network. With the increase of W_bran_, the color of the steamed bread developed by the TBBF–WF blend gradually darkened and yellowed, the specific volume declined, and its hardness, gumminess, and chewiness ascended gradually. The appropriate addition of TBBF (W_bran_ = 10% and 30%) was beneficial to cell diameter and volume of steamed bread, but the further rise of W_bran_ would destroy its gas retention ability. The predicted glycemic index (pGI) of steamed bread declined significantly with the increasing W_bran_.

## 1. Introduction

Tartary buckwheat (*Fagopyrum tataricum* L.), a dicotyledonous crop of the Polygonaceae family, is widely distributed in China, Russia, South Korea, Japan, Europe, and other regions [1,2]. For its high nutritional and medicinal value, it has received a lot of attention as a health food [3,4]. Compared with other cereals such as wheat, rice and corn, Tartary buckwheat is not only rich in protein, lipid, starch, dietary fiber, minerals, and vitamins, but also abundant in polyphenols represented by rutin, which play a positive role in the prevention and treatment of chronic and age-related diseases, such as hypertension, hyperlipidemia, hyperglycemia, and cardiovascular diseases [5,6,7]. Tartary buckwheat bran (TBB) is the main co-product of Tartary buckwheat processing, which is rich in rutin and has a variety of biological activities [8,9]. Yet, at present TBB is mainly used as animal feed, resulting in great waste; thus, its comprehensive development needs to be carried out urgently. The current research on TBB is mainly focused on the separation, purification, identification and bioactivities of polyphenols in TBB [10,11,12], but there are few studies on the application of TBB in the development of staple foods.

Steamed bread is a traditional staple food in China, accounting for 30% of the country’s wheat consumption [13]. With the development of mechanization and industrialization, steamed bread production technology is gradually enhanced, and the variety of products is increasing, which is favored by consumers. Although commercial steamed bread is mainly made with refined wheat flour (WF) with good sensory quality and cooking quality, the use of WF leads to problems such as excess of major nutrients and lack of micronutrients, which reduces the overall nutritional value of steamed bread [14,15]. TBB possesses a variety of functional ingredients. Replacing WF with TBB can not only meet the needs of consumers for healthy diet, but also provide theoretical reference for the development of functional staple foods. In view of this, the chemical composition and physicochemical properties of Tartary buckwheat flour (TBF), Tartary buckwheat bran flour (TBBF), and Tartary buckwheat core flour (TBCF) were compared. On this basis, the processing characteristics of TBBF–WF flour blend were systematically investigated, and the effect of the weight proportion of TBBF in the blend (W_bran_) on the appearance, texture and starch digestion of steamed bread were clarified.

## 2. Materials and Methods

### 2.1. Materials and Chemicals

Tartary buckwheat (variety, Jinqiao 2) was purchased from the Institute of Crop Science, Shanxi Agricultural University (Taiyuan, China). WF was the product of China Oil & Foodstuffs Corporation (Beijing, China). The dry yeast was from Angel Yeast Co., Ltd. (Yichang, China). Rutin, 1,1-diphenyl-2-picrylhydrazyl (DPPH) and 2,2′-azinobis(3-ethylbenzothiazoline-6-sulfonic acid) diammonium salt (ABTS) were bought from Sigma-Aldrich (St. Louis, MO, USA). The other chemicals were of analytical grade.

### 2.2. Preparation of TBF, TBBF and TBCF

**Figure 1:**
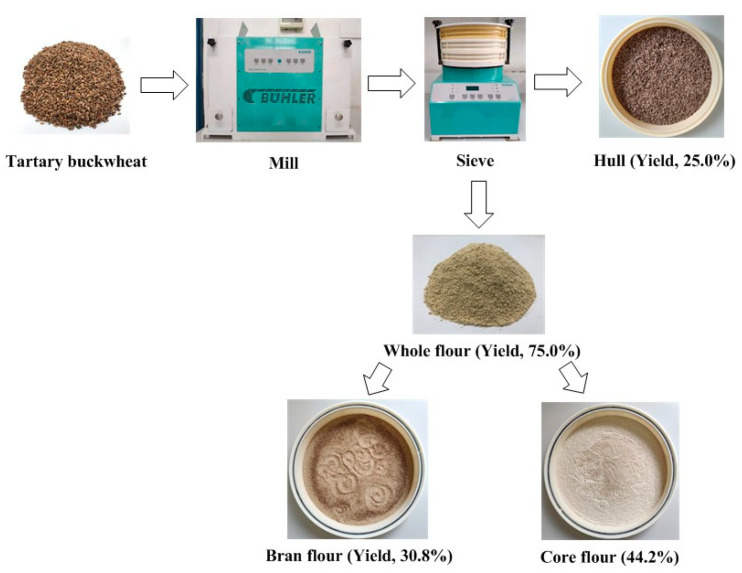
Preparation of whole flour, core flour, and bran flour of Tartary buckwheat.

As shown in Figure 1, the cleaned Tartary buckwheat seeds were ground with a Bühler ALMB laboratory mill (Wuxi, China), then sifted at the speed of 300 rpm for 200s by a Bühler ALMC sieve shaking machine (Wuxi, China) with 26GG and 7XX sieves. The material on the 26GG sieve (-/26GG) was Tartary buckwheat hull, and the material under the 26GG sieve (26GG/-) was TBF. TBF could be further screened into TBBF (26GG/7XX) and TBCF (7XX/-). TBF, TBBF, and TBCF were crushed by a grinder, sifted through 100 mesh and preserved for the following experiments.

### 2.3. Routine Composition Determination

The routine composition determination of TBF, TBBF, and TBCF was carried out according to Chinese national standard methods. The moisture content was measured by the oven-drying method (GB 5009.3-2016). The mineral content was determined based on the dry ashing method with a muffle furnace (GB 5009.4-2016). The measurement of protein content was performed on a Kjeldahl apparatus (GB 5009.5-2016). The lipid was extracted by ethyl ether in a Soxhlet apparatus and determined according to GB 5009.6-2016. The starch content was measured using the polarimetric method (GB/T 20194-2018). The above results were expressed on a wet basis.

### 2.4. Determination of Rutin Content

The sample (3 g) was extracted with 180 mL of 80% ethanol at 80 °C for 7 h, and then filtered. The supernatant was diluted to 250 mL for HPLC analysis. HPLC measurement was performed on an Agilent 1260 HPLC system (Santa Clara, CA, USA) with an Agilent ZORBAX SB-C18 column (4.6 × 150 mm, 5 μm particle size). The mobile phase consisted of methanol and water (6:4, *v*/*v*) with the flow rate of 1.0 mL/min. The injection volume was 10 μL while the detection wavelength was set at 265 nm. The column temperature was set at 30 °C. The chromatographic data were recorded and processed by Agilent OpenLAB ChemStation software (Santa Clara, CA, USA). The rutin content was determined based the external standard method [16].

### 2.5. Determination of Fatty Acid Composition

According to the method of Ji et al. [17], the sample (5 g) was extracted with 150 mL of n-hexane at 60 °C for 5 h, and then filtered. The filtrate was concentrated under vacuum at 45 °C to remove n-hexane. Then, the methylation of residue was achieved with BF_3_-methanol reagent for GC measurement. GC analysis was performed on an Agilent 7890B gas chromatograph (Santa Clara, CA, USA) equipped with a flame ionization detector. The sample solution (1 μL) was separated by a HP-88 capillary column (100 m × 0.25 mm i.d. × 0.20 μm f.t.) with the split ratio of 50:1. High purity nitrogen was used as the carrier gas with a flow rate of 1 mL/min. Column temperature program was 140 °C (5 min) isotherm, then increased to 240 °C at the rate of 4 °C/min and was held for 10 min. The temperatures of injector and detector were 260 °C and 280 °C, respectively. The fatty acid composition was determined by comparing with standard fatty acid methyl esters.

### 2.6. Determination of Amino Acid Composition

According to the method of Ji et al. [17], 40 mg of sample was defatted with n-hexane and petroleum ether and moved into the hydrolysis tube. Then, 10 mL of 6 mol/L hydrochloric acid and 3-4 drops of freshly distilled phenol were added. The hydrolysis tube was filled with nitrogen and tightly covered, heated in an oven at 110 °C for 22 h. After hydrolysis, the suspension was filtered, and the tube was washed several times with ultra-pure water. The obtained hydrolysate was diluted to 50 mL with ultrapure water. Then, 1 mL of the above solution was dried under vacuum at 40 °C. The residue was re-dissolved in 1 mL ultra-pure water and dried again. This process was repeated twice. Finally, the residue was re-dissolved with 1 mL PBS (pH 2.2) and passed through the 0.22-μm filter membrane. Its amino acid composition was determined by an amino acid analyzer (Sykam, Eresing, Germany). The results were expressed as the percentage of each amino acid in the weight of the raw material.

### 2.7. Evaluation of Antioxidant Activity

The sample (3 g) was extracted with 180 mL of 80% ethanol at 80 °C for 7 h, and then filtered. The filtrate was concentrated at 40 °C under vacuum and lyophilized by an Alpha 1–4 freezer dryer (Christ, Osterode, Germany), and the extract was collected for the DPPH and ABTS radical scavenging assay.

The DPPH scavenging activity of the sample was evaluated by the method of Ngamsuk et al. [18], 2 mL of the sample ethanol solution at different concentrations and 2 mL of 2 × 10^−4^ mol/L DPPH ethanol solution were mixed and incubated for 0.5 h at 25 °C. Then, the absorbance of sample solution at 517 nm (A_sample_) was read. The control was a mixture of 2.0 mL DPPH solution and 2.0 mL ethanol, and its absorbance at 517 nm (A_control_) was also measured. The DPPH radical scavenging activity could be calculated based on the following formula:

(1) $$\mathrm{DPPH}\;\mathrm{radical}\;\mathrm{scavenging}\;\mathrm{activity}\;\left(\%\right)=\frac{A_{\mathrm{control}}-A_{\mathrm{sample}}}{A_{\mathrm{control}}}\times 100\%$$

The ABTS scavenging activity of the sample was evaluated by the method of Fogarasi et al. [19]. First, 100 mL of 7 mM ABTS solution and 1.75 mL of 2.45 mM potassium persulfate solution was mixed, and incubated at 25 °C for 12 h. The above mixture was diluted with PBS (0.05 M, pH7.4) until its absorbance at 734 nm was 0.70 ± 0.02, which was collected as the ABTS test solution. 0.15 mL of the sample ethanol solution at different concentrations and 2.85 mL of ABTS test solution were mixed and incubated for 10 min at 25 °C. Then, its absorbance at 734 nm (A_sample_) was read. The absorbance of the mixture of 0.15 mL ethanol and 2.85 mL ABTS test solution at 734 nm was also measured as A_control_. The ABTS radical scavenging activity could be calculated based on the following formula:

(2) $$\mathrm{ABTS}\;\mathrm{radical}\;\mathrm{scavenging}\;\mathrm{activity}\;\left(\%\right)=\frac{A_{\mathrm{control}}-A_{\mathrm{sample}}}{A_{\mathrm{control}}}\times 100\%$$

### 2.8. Determination of Water Absorption Index and Water Solubility Index

According to the method of Heo et al. [20], the water absorption index (WAI) and water solubility index (WSI) of TBF, TBBF, and TBCF were evaluated. The sample (1.5 g) was put into a 50 mL centrifuge tube, mixed with 20 mL distilled water, and oscillated violently until it was completely dispersed to form a suspension system. The mixture was incubated at 30 °C for 30 min, oscillating continuously, and then centrifuged at 6000 rpm for 15 min. Finally, the supernatant was poured into an aluminum box and dried at 105 °C to constant weight. The WAI and WSI could be calculated based on the following equations:

(3) $$WSI\;(\%)=\frac{m_{1}}{m_{0}}\times 100\%$$

(4) $$WAI\;(\%)=\frac{m_{2}}{m_{0}}\times 100\%$$

where *m*_0_, *m*_1_ and *m*_2_ are the sample mass, the solid mass in the supernatant and the precipitation mass in the centrifuge tube, respectively.

### 2.9. Determination of Dough Rheological Properties

The dough rheological properties of TBF, TBBF, TBCF, and TBBF-WF flour blend (W_bran_ = 0, 10, 30, 50, 70, and 90%) were measured by a Mixolab 2 apparatus (Chopin, Paris, France) based on the “Chopin+” protocol. The dough weight was set at 75 g, and the amount of water added was corrected based on 14% moisture content, which made the dough produce the torque of 1.1 ± 0.5 Nm at the mixing speed of 80 rpm. The protocol was run for a total of 45 min, including three stages: (1) Constant temperature stage, the dough was mixed at the speed of 80 rpm at 30 °C for 8 min; (2) Heating stage, the dough was heated to 90 °C at a rate of 4 °C/min in 15 min and kept for 7 min; (3) Cooling stage, the dough was cooled to 50 °C at a rate of 4 °C/min in 10 min and held for 5 min.

### 2.10. Preparation of Steamed Bread

The steamed bread containing TBBF-WF flour blend (W_bran_ = 0, 10, 30, 50, 70, and 90%) were prepared according to Chinese Standard GB/T 35991-2018. Dry yeast (1 g, 1%) was dissolved in water (38 °C). Then, 100 g of flour and the yeast solution was mixed in a K5SS blender (Whirlpool, Benton Harbor, MI, USA) for 7 min until the dough was formed. The dough was incubated in a Shengheng FJX-13 fermentation tank (Guangzhou, China) for 1 h at 37 °C and 85% RH. The obtained dough was rolled 10 times by a Fuxing DMT-10B dough sheeter with 5-mm roller gap (Longkou, China). The treated dough was divided into two pieces. Each piece was shaped by hand to yield a smooth dough ball, placed in a steamer and proofed for 0.5 h at 37 °C and 85% RH. The dough pieces were steamed for 25 min and cooled for 60 min for the following measurements.

### 2.11. Quality Evaluation of Steamed Bread

The color of steamed bread was determined using a CR-400 colorimeter (Konica-Minolta, Osaka, Japan) using the L*, a*, and b* color scale. Its volume was measured using rapeseed displacement. Then, the corresponding specific volume was calculated by dividing volume by weight. The textural properties of steamed bread were evaluated based on the TPA mode by a TA-XT Plus texture analyzer (Stable Micro Systems, Surrey, UK) equipped with a P/36R probe. The pretest speed, test speed and post-test speed of probe were 3.0 mm/s, 1.0 mm/s, and 1.0 mm/s, respectively. The compression strain was set at 30% while the auto trigger force was 5.0 g. The data were recorded and processed by Exponent 6.1 software. The image of steamed bread slice was analyzed by a C-Cell imaging system (Calibre Control International Ltd., Warrington, UK), which could provide the detailed structural information, such as slice area, cell diameter, cell volume and so on.

### 2.12. In Vitro Digestion Method

The in vitro digestion of steamed bread was performed on a NutriScan GI20 glycemic index analyzer at 37 °C. The freeze-dried and crushed steamed bread containing 50 mg starch was mixed with 2 mL simulated saliva solution, and the peristaltic movement of oral digestion was simulated by a rotor. After 5 min, 5 mL simulated gastric solution was added to the above solution and reacted for 1.5 h. After the pH value of the solution was adjusted, it was mixed with 5 mL simulated intestinal fluid and hydrolyzed for 4 h. The released glucose was monitored by the built-in glucose analyzer, and the predicted glycemic index (pGI) was calculated automatically [21].

### 2.13. Statistical Analysis

The experimental results were expressed as the average ± standard deviation (*n* = 3). The statistical comparison was based on the Tukey method with a confidence level of 95% using the SPSS 18 software package (SPSS Inc., Chicago, IL, USA).

## 3. Results and Discussion

### 3.1. Chemical Composition

As shown in Table 1, there were significant differences in the chemical compositions of TBF, TBCF, and TBBF (*p* < 0.05). The moisture content and starch content of TBBF were lower than those of TBF and TBCF, while its mineral, protein, lipid, and rutin contents were much higher than those of TBF and TBCF. As a flavonoid, rutin has many health-promoting functions, such as antioxidant, anti-inflammatory, anti-tumor, and antiviral activities, among others [22,23]. The rutin content of TBBF was as high as 3.27%, which was higher than that of common cereal flour. The lipid content of TBBF was also superior to that of TBF, which was more than 3 times that of TBCF, and its fatty acid composition was consistent with that of TBF and TBCF. It was rich in unsaturated fatty acids such as oleic acid (C18:1), linoleic acid (C18:2), linolenic acid (C18:3), and its total unsaturated fatty acid composition was more than 81% (Table 2), which is beneficial for reducing blood lipids, prevent atherosclerosis, and maintaining cognitive ability [24]. Similarly, due to the high protein content of TBBF, the content of various amino acids in TBBF was significantly higher than that in TBF and TBCF. And it was rich in the essential amino acids such as lysine, threonine, and valine (Table 3).

### 3.2. Antioxidant Activities

**Figure 2:**
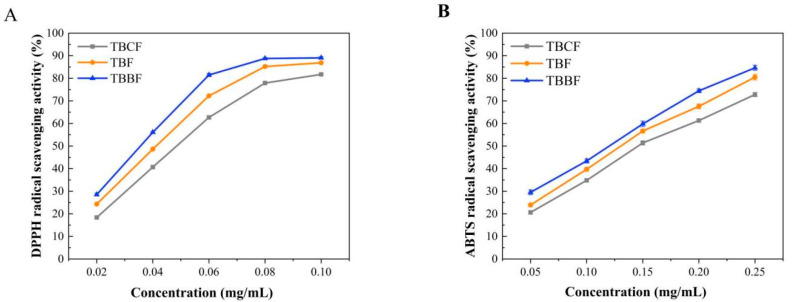
DPPH (**A**) and ABTS (**B**) radical scavenging activities of TBF, TBCF, and TBBF (TBF: Tartary buckwheat flour; TBCF: Tartary buckwheat core flour; TBBF: Tartary buckwheat bran flour).

Reactive oxygen species (reactive oxygen species, ROS) are mainly produced from the mitochondrial respiratory chain in cells under oxidative stress, which play an important role in a variety of physiological and pathological processes [25]. Many reports confirm that under normal circumstances, the ROS in the organism always maintains a very low level of balance, but the level of ROS in mitochondria in many diseases such as cardiovascular disease, diabetes, tumor, and inflammation is much higher than that in normal cells, and plant-derived antioxidants can effectively maintain the balance of ROS, and reduce oxidative stress and age-related diseases [26,27]. DPPH and ABTS are both relatively stable synthetic free radicals. When antioxidants are present, the color of their solutions will gradually fade, and the activity of antioxidants can be reflected according to the change of absorbance at 517 and 734 nm. In this study, the DPPH and ABTS radical scavenging capacities of the extracts of TBF, TBCF, and TBBF were systematically evaluated (Figure 2). All the extracts exhibited strong DPPH and ABTS scavenging activities in a concentration-dependent manner. At the same concentration, there were significant differences in scavenging performance (*p* < 0.05), their activities followed this order: TBBF > TBF > TBCF. The high antioxidant activity of TBBF should be attributed to its high rutin content.

### 3.3. Dough Properties

**Figure 3:**
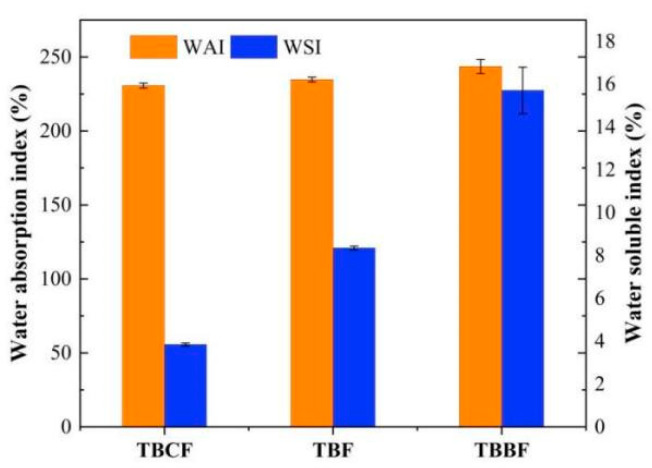
Water absorption index (WAI) and Water soluble index (WSI) of TBF, TBCF and TBBF (TBF: Tartary buckwheat flour; TBCF: Tartary buckwheat core flour; TBBF: Tartary buckwheat bran flour).

WAI represents the water-holding capacity of the sample, while WSI reflects the solubility of the sample in water [28]. It could be observed from Figure 3 that there is no significant difference in WAI among the samples, but there was a significant difference in their WSI (*p* < 0.05) with the order of TBBF > TBCF > TBF. The outstanding performance of TBBF might be attributed to its high protein content.

**Figure 4:**
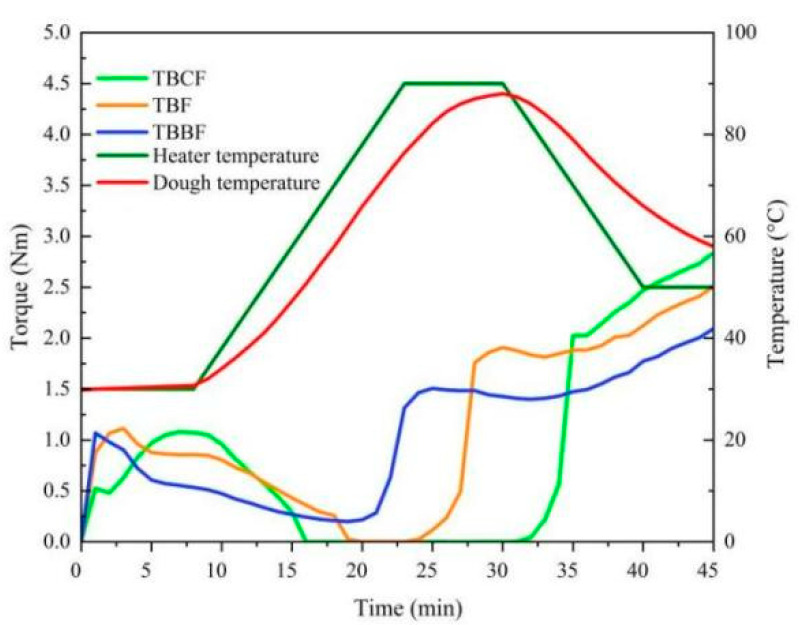
Mixolab profiles of TBF, TBCF and TBBF (TBF: Tartary buckwheat flour; TBCF: Tartary buckwheat core flour; TBBF: Tartary buckwheat bran flour).

The dough properties of TBF, TBCF, and TBBF were measured with the Mixolab^®^ protocol. As shown in Figure 4 and Table 4, different samples had different dough torque curves. At the initial stage, the dough formation time of TBBF was significantly lower than that of TBF and TBCF, which could be due to the fact that its high protein content could accelerate dough formation. During the heating process, the torque C2 values of TBF and TBCF were 0, suggesting that their protein weakening degrees were very large, and the dilution effect caused by them reduced the viscosity of the dough. The hardness and stickiness of the gelatinized dough of TBBF was higher than that of TBCF and less than that of TBF since its torque C3 (Peak viscosity of gelatinization) was between TBF and TBCF. When the temperature decreased, the torque C5 of TBBF was obviously lower than that of TBF and TBCF, confirming that its starch retrogradation was not easy to occur.

**Figure 5:**
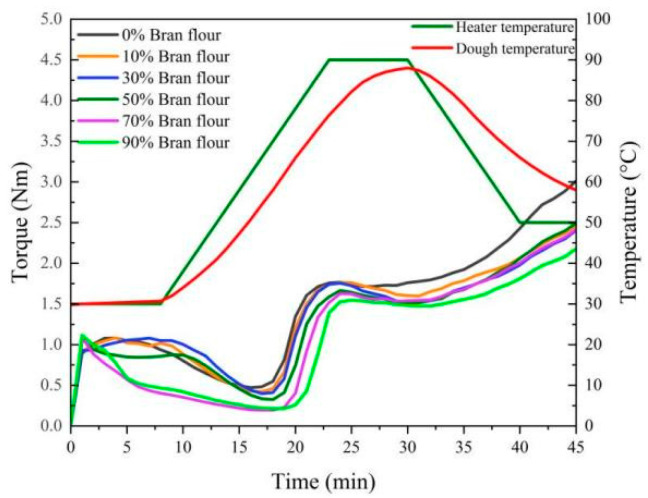
Effect of W_bran_ on the mixolab curves of mixed dough (TBF: Tartary buckwheat flour; TBCF: Tartary buckwheat core flour; TBBF: Tartary buckwheat bran flour; W_bran_: the weight proportion of TBBF in the blend four).

TBBF was rich in rutin and protein, so it possessed excellent antioxidant activity and dough rheological properties, so it can be used in the development of functional staple foods. Herein, the dough rheological properties of TBBF–-WF flour blend were investigated systematically. The ratio of TBBF in the blend (W_bran_) had a significant effect on the torque curve and parameters of dough (Figure 5 and Table 5). With the increase of W_bran_, the water absorption of dough decreased gradually, which may be due to the fact that the water absorption of TBBF was slightly lower than that of WF. When W_bran_ increased from 0% to 30%, the development time and stability time of the dough increased significantly, and reached the maximum at 30%, confirming that the dough had the best endurance to mixing. When W_bran_ was more than 30%, the development time and stability time of the dough decreased greatly, and the dough properties decreased sharply. With the addition of TBBF, the protein weakening degree (C2) also decreased rapidly, indicating that TBBF caused the dilution effect of dough and weakened the gluten network. In addition, when W_bran_ increased from 0% to 30%, there was no significant difference in C3. When it increased from 50% to 90%, C3 decreased significantly, suggesting that the hardness and stickiness of the dough decreased gradually after gelatinization. C3-C2 (Gelatinization degree) and β (Gelatinization rate) reached the maximum when W_bran_ was 70%, at this time, the gelatinization performance was the highest, and the gelatinization speed was the fastest. The dough developed at W_bran_ = 90% exhibited the highest thermal stability and resistance to starch retrogradation since it had the smallest C3-C4 and C5-C4 values.

### 3.4. Steamed Bread

**Figure 6:**
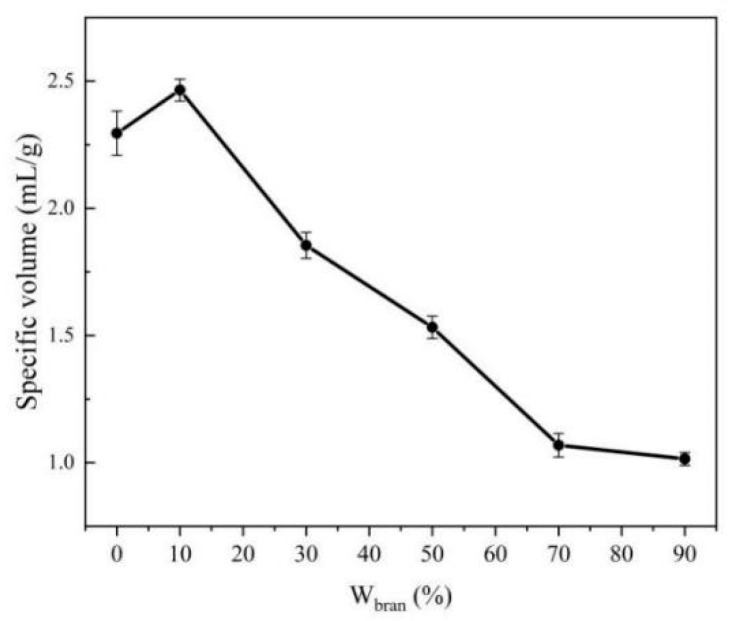
Effect of W_bran_ on the specific volume of steamed bread (W_bran_: the weight proportion of TBBF in the blend four).

The color and specific volume of steamed bread is an indispensable part that determines the sensory quality of steamed bread, which directly affects the acceptance of consumers [29]. The effect of W_bran_ on the color difference of steamed bread was investigated (Table 6). With the increase of W_bran_, L* decreased significantly, and the values of a* and b* increased at first and then decreased, indicating that the color of the steamed bread gradually darkened and yellowed, which could be related to the yellowish green of TBBF itself. Figure 6 demonstrated the effect of W_bran_ on the specific volume of steamed bread. With the increase of W_bran_, the specific volume of steamed bread declined gradually. For the steamed breads developed at W_bran_ = 70% and 90%, their specific volumes had no difference (*p* > 0.05), which was close to 1. It may be due to the dilution effect of TBBF on gluten protein, which hindered the formation of gluten network, resulting in the decrease of dough expansion and the reduction of steamed bread volume during fermentation.

Chewiness and hardness are the important indexes to reflect the quality of steamed bread. The chewiness, hardness, and gumminess of steamed bread are negatively correlated with the quality of steamed bread, while the resilience and springiness are positively correlated with the quality of steamed bread [13]. Table 7 exhibited the effect of W_bran_ on the texture of steamed bread. With the addition of TBBF, the hardness, gumminess, and chewiness of steamed bread gradually increased, while the springiness increased at first and then decreased, and there was no significant change after the addition of 50% TBBF. This may be due to the dilution of gluten concentration in dough caused by TBBF, which destroyed the gluten network to a certain extent, and the structure of steamed bread was not fluffy enough, leading to the increase of hardness and chewiness and decrease of springiness of steamed bread. The change trend of cohesiveness and resilience was similar, the addition of TBBF had no significant change at 0% and 10%, but cohesiveness and resilience reduced with the increase of W_bran_, and there was no significant change when W_bran_ exceeded 50%. This may be due to the fact that the addition of TBBF affected the formation of gluten network structure, resulting in incomplete gluten network structure formed after dough fermentation, and it was difficult to return to its original state after compression deformation, so the cohesiveness and resilience descended.

**Figure 7:**
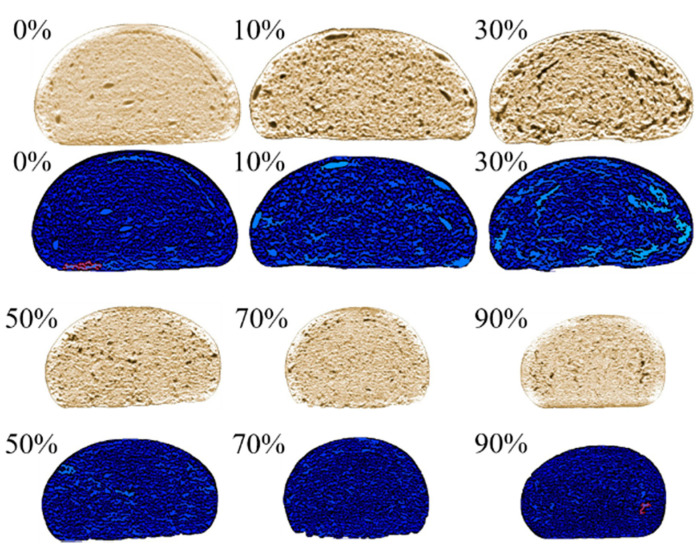
Effect of W_bran_ on the raw and cell images of steamed bread slices (W_bran_: the weight proportion of TBBF in the blend four).

C-Cell image analyzer is a quality control system of fermented flour products based on computer recognition technology. Through image processing of sample slices, it can obtain the characteristic parameters of cells and slices, and comprehensively evaluate the internal texture structure of products [30]. Figure 7 shows the C-Cell images of steamed bread with different W_bran_ values, the corresponding parameters were summarized in Table 8. With the increase of W_bran_, the slice area, brightness, and cell area of steamed bread decreased, cell contrast, cell number, cell elongation, and cell density decreased at first and then increased, while cell diameter, wall thickness, cell volume, and coarse cell volume increased at first and then decreased. The addition of TBBF at the level of 10% and 30% could reduce the texture of steamed bread. When W_bran_ ≥ 50%, the air-holding property of the dough decreased during the fermentation process, leading to a tighter tissue and a sandy interior of the steamed bread. After 10%, the addition of TBBF could make the cell wall thinner, improve cell contrast, and weaken the gloss of steamed bread.

### 3.5. pGI

**Figure 8:**
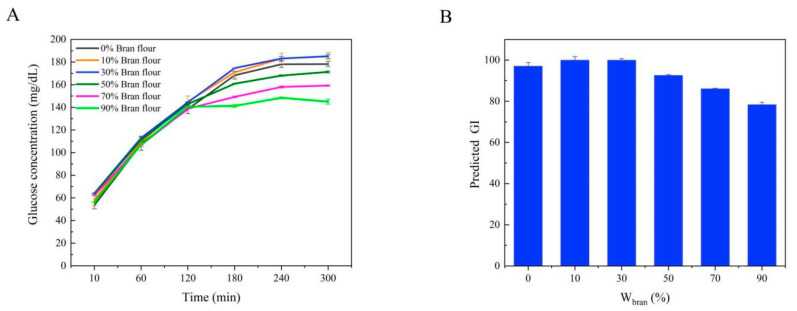
Effect of Wbran on the starch hydrolysis (**A**) and predicted GI value (**B**) of steamed bread (W_bran_: the weight proportion of TBBF in the blend four).

The glycemic index (GI) of food is used to reflect the effect of carbohydrates in food on blood glucose concentration [31]. Foods with high GI can be digested quickly and have a high absorption rate after entering the gastrointestinal tract, resulting in a rapid rise in blood glucose concentration. Foods with low GI stay in the gastrointestinal tract for a long time and have a low absorption rate, which leads to a slow rise in blood glucose concentration. Studies have shown that low-GI foods can improve blood glucose control in patients with diabetes and play a positive role in the prevention of cardiovascular diseases [32]. However, the determination of GI value is more complicated, and the NutriScan GI20 glycemic index analyzer can real-time track the hydrolysis of starch during simulated digestion of food and convert the results into the predicted GI value (pGI). The effect of W_bran_ on starch hydrolysis during steamed bread digestion is shown in Figure 8A.

With the progress of digestion, the starch hydrolysis of steamed bread with W_bran_ of 0%, 10% and 30% was carried out quickly before 180 min and stabilized after 180 min. The starch hydrolysis of steamed bread developed at W_bran_ = 50, 70 and 90% also increased rapidly before 120 min, and the growth rate was slow and stable after 120 min. At 300 min, the starch hydrolysis of steamed bread developed at W_bran_ = 90% was the lowest. Based on the whole digestion process, the pGI values of steamed breads with different W_bran_ values could be obtained (Figure 8B). With the increase of W_bran_, the pGI value of steamed bread decreased gradually. The mechanism of low starch digestion of the steamed bread developed by TBBF–WF blend may be attributed to the following reasons: (1) TBBF is rich in polyphenols represented by rutin, which interact with starch to form the complex that can reduce the digestibility of starch; on the other hand, polyphenols can also combine with digestive enzymes such as α-amylase and α-glucosidase to further reduce the hydrolysis rate of starch; (2) TBBF is rich in lipids, and the starch-lipid complex formed can resist the effect of digestive enzymes and retard the hydrolysis of starch; (3) TBBF is rich in proteins, which can wrap starch to a certain extent. The protein attached to the surface of starch granules can hinder the contact between digestive enzymes and starch and delay starch hydrolysis.

## 4. Conclusions

In this study, TBF, TBCF, and TBBF were prepared by mechanical grinding combined with sieving, and their chemical composition, antioxidant activity, and dough rheological properties were compared. It was found that TBBF was rich in rutin and protein, so its antioxidant performance and dough rheological properties were the best, and it could be mixed with WF for steamed bread production. Also, the dough developed at W_bran_ = 30% had the best kneading resistance. When W_bran_ was more than 30%, it would cause dilution effect and weaken the dough rheological properties. With the increase of W_bran_, the color of steamed bread darkened and yellowed, the specific volume gradually decreased, its hardness, adhesion and chewiness gradually increased, while the pGI value of steamed bread decreased significantly. Our results indicate that TBBF is a new food source with antioxidant and hypoglycemic activities, which can be used in the development of new functional staple foods.

## Figures and Tables

**Table 1:** Chemical composition of TBF, TBCF, and TBBF.

	TBF	TBCF	TBBF
Water (%)	9.96 ± 0.02 ^b^	10.78 ± 0.03 ^a^	9.55 ± 0.03 ^c^
Mineral (%)	1.79 ± 0.01 ^b^	0.93 ± 0.01 ^c^	2.96 ± 0.01 ^a^
Lipid (%)	2.62 ± 0.02 ^b^	1.15 ± 0.01 ^c^	4.76 ± 0.02 ^a^
Starch (%)	68.28 ± 0.11 ^b^	81.94 ± 0.15 ^a^	49.32 ± 0.10 ^c^
Protein (%)	14.64 ± 0.27 ^b^	8.07 ± 0.06 ^c^	23.33 ± 0.14 ^a^
Rutin (%)	1.33 ± 0.01 ^b^	0.04 ± 0.01 ^c^	3.27 ± 0.36 ^a^

Different superscript lowercase letters in the same row mean significantly differences (*p* < 0.05); TBF, TBCF, and TBBF represent Tartary buckwheat flour, Tartary buckwheat core flour and Tartary buckwheat bran flour, respectively.

**Table 2:** Fatty acid composition (%) of TBF, TBCF, and TBBF.

	TBF	TBCF	TBBF
C16:0	21.29 ± 7.46 ^a^	13.92 ± 0.06 ^a^	14.11 ± 0.18 ^a^
C16:1	0.13 ± 0.03 ^b^	0.09 ± 0.01 ^c^	0.18 ± 0.00 ^a^
C18:0	2.04 ± 0.20 ^b^	2.47 ± 0.03 ^a^	2.11 ± 0.04 ^b^
C18:1	35.68 ± 3.39 ^b^	40.83 ± 0.85 ^a^	38.53 ± 0.03 ^ab^
C18:2	34.73 ± 3.27 ^a^	36.25 ± 0.84 ^a^	38.34 ± 0.19 ^a^
C18:3	3.60 ± 0.37 ^a^	3.68 ± 0.06 ^a^	3.98 ± 0.02 ^a^
C20:0	1.30 ± 0.13 ^a^	1.42 ± 0.01 ^a^	1.42 ± 0.01 ^a^
C22:0	1.23 ± 0.13 ^a^	1.36 ± 0.03 ^a^	1.33 ± 0.01 ^a^

Different superscript lowercase letters in the same row mean significantly differences (*p* < 0.05); TBF, TBBF and TBCF represent Tartary buckwheat flour, Tartary buckwheat bran flour and Tartary buckwheat core flour, respectively.

**Table 3:** Amino acid composition (%) of TBF, TBCF, and TBBF.

	TBF	TBCF	TBBF
Asp	0.95 ± 0.28 ^b^	0.55 ± 0.10 ^c^	1.82 ± 0.14 ^a^
Thr	0.38 ± 0.12 ^b^	0.23 ± 0.04 ^b^	0.70 ± 0.07 ^a^
Ser	0.52 ± 0.16 ^b^	0.30 ± 0.06 ^c^	0.99 ± 0.06 ^a^
Glu	1.88 ± 0.60 ^b^	0.96 ± 0.15 ^c^	3.71 ± 0.29 ^a^
Gly	0.61 ± 0.18 ^b^	0.34 ± 0.06 ^c^	1.14 ± 0.11 ^a^
Ala	0.45 ± 0.14 ^b^	0.28 ± 0.06 ^b^	0.80 ± 0.08 ^a^
Cys	0.09 ± 0.03 ^a^	0.03 ± 0.01 ^b^	0.11 ± 0.01 ^a^
Val	0.55 ± 0.16 ^b^	0.33 ± 0.05 ^c^	0.99 ± 0.09 ^a^
Met	0.17 ± 0.04 ^b^	0.10 ± 0.01 ^b^	0.33 ± 0.05 ^a^
Ile	0.40 ± 0.11 ^b^	0.25 ± 0.05 ^b^	0.71 ± 0.09 ^a^
Leu	0.64 ± 0.20 ^b^	0.41 ± 0.08 ^b^	1.17 ± 0.08 ^a^
Tyr	0.38 ± 0.12 ^b^	0.17 ± 0.04 ^c^	0.63 ± 0.07 ^a^
Phe	0.52 ± 0.17 ^b^	0.30 ± 0.03 ^b^	0.90 ± 0.08 ^a^
His	0.40 ± 0.10 ^b^	0.22 ± 0.04 ^c^	0.77 ± 0.07 ^a^
Lys	0.57 ± 0.18 ^b^	0.34 ± 0.05 ^b^	1.04 ± 0.10 ^a^
Arg	1.01 ± 0.31 ^b^	0.46 ± 0.08 ^c^	2.00 ± 0.15 ^a^
Pro	0.37 ± 0.09 ^b^	0.26 ± 0.02 ^b^	0.77 ± 0.10 ^a^

Different superscript lowercase letters in the same row mean significantly differences (*p* < 0.05); TBF, TBCF and TBBF represent Tartary buckwheat flour, Tartary buckwheat core flour and Tartary buckwheat bran flour, respectively.

**Table 4:** Parameters values of Mixolab curves of all samples.

	TBF	TBCF	TBBF
Water absorption (%)	56.9 ± 0.3 ^b^	64.0 ± 0.1 ^a^	55.0 ± 0.1 ^c^
Development time (min)	2.843 ± 0.090 ^b^	6.807 ± 0.552 ^a^	0.920 ± 0.053 ^c^
Thermal stability (min)	2.300 ± 0.100 ^b^	5.500 ± 0.100 ^a^	2.433 ± 0.153 ^b^
C1 (Nm)	1.102 ± 0.033 ^a^	1.094 ± 0.022 ^a^	1.099 ± 0.035 ^a^
C2 (Nm)	0.000 ± 0.000 ^b^	0.000 ± 0.000 ^b^	0.200 ± 0.001 ^a^
C3 (Nm)	1.838 ± 0.055 ^b^	2.069 ± 0.132 ^a^	1.482 ± 0.033 ^c^
C4 (Nm)	1.711 ± 0.036 ^b^	1.891 ± 0.124 ^a^	1.389 ± 0.007 ^c^
C5 (Nm)	2.531 ± 0.072 ^b^	2.832 ± 0.045 ^a^	2.070 ± 0.036 ^c^
C3-C2 (Nm)	1.838 ± 0.055 ^b^	2.069 ± 0.132 ^a^	1.282 ± 0.034 ^c^
C3-C4 (Nm)	0.127 ± 0.064 ^a^	0.178 ± 0.071 ^a^	0.089 ± 0.033 ^a^
C5-C4 (Nm)	0.821 ± 0.075 ^a^	0.941 ± 0.086 ^a^	0.677 ± 0.036 ^b^
α (Nm/min)	−0.007 ± 0.002 ^a^	−0.110 ± 0.010 ^c^	−0.039 ± 0.006 ^b^
β (Nm/min)	0.005 ± 0.001 ^c^	0.008 ± 0.002 ^b^	0.603 ± 0.082 ^a^
γ (Nm/min)	0.017 ± 0.021 ^a^	0.395 ± 0.482 ^a^	−0.020 ± 0.010 ^a^

Different superscript lowercase letters in the same row mean significantly differences (*p* < 0.05); TBF, TBCF and TBBF represent Tartary buckwheat flour, Tartary buckwheat core flour and Tartary buckwheat bran flour, respectively.

**Table 5:** Effect of W_bran_ on parameters values of Mixolab curves of mixed dough.

W_bran_ (%)	0	10	30	50	70	90
Water absorption (%)	59.3 ± 0.2 ^a^	58.7 ± 0.1^b^	57.3 ± 0.3 ^c^	54.8 ± 0.2 ^f^	55.4 ± 0.1 ^e^	55.9 ± 0.2 ^d^
Development time (min)	3.290 ± 0.082 ^c^	3.550 ± 0.130 ^b^	6.990 ± 0.225 ^a^	0.833 ± 0.015 ^d^	0.860 ± 0.026 ^d^	0.967 ± 0.015 ^d^
Thermal stability (min)	6.567 ± 0.289 ^c^	7.933 ± 0.321 ^b^	10.067 ± 0.513 ^a^	1.467 ± 0.058 ^de^	1.067 ± 0.058 ^e^	1.900 ± 0.346 ^d^
C1 (N·m)	1.091 ± 0.011 ^b^	1.097 ± 0.021 ^ab^	1.064 ± 0.029 ^b^	1.100 ± 0.032 ^ab^	1.098 ± 0.009 ^ab^	1.138 ± 0.017 ^a^
C2 (N·m)	0.465 ± 0.010 ^a^	0.423 ± 0.006 ^b^	0.388 ± 0.010 ^c^	0.325 ± 0.005 ^d^	0.192 ± 0.002 ^f^	0.210 ± 0.002 ^e^
C3 (N·m)	1.758 ± 0.023 ^a^	1.787 ± 0.019 ^a^	1.767 ± 0.009 ^a^	1.683 ± 0.005 ^b^	1.635 ± 0.009 ^c^	1.525 ± 0.035 ^d^
C4 (N·m)	1.670 ± 0.060 ^a^	1.617 ± 0.034 ^a^	1.491 ± 0.012 ^b^	1.485 ± 0.010 ^b^	1.502 ± 0.007 ^b^	1.447 ± 0.022 ^b^
C5 (N·m)	2.937 ± 0.089 ^a^	2.532 ± 0.061 ^b^	2.379 ± 0.056 ^c^	2.513 ± 0.006 ^b^	2.401 ± 0.027 ^c^	2.158 ± 0.040 ^d^
C3-C2 (N·m)	1.293 ± 0.014 ^c^	1.364 ± 0.013 ^b^	1.379 ± 0.004 ^b^	1.358 ± 0.001 ^b^	1.443 ± 0.011 ^a^	1.315 ± 0.034 ^c^
C3-C4 (N·m)	0.089 ± 0.038 ^d^	0.170 ± 0.018 ^bc^	0.275 ± 0.012 ^a^	0.198 ± 0.014 ^b^	0.133 ± 0.014 ^c^	0.078 ± 0.015 ^d^
C5-C4 (N·m)	1.268 ± 0.053 ^a^	0.915 ± 0.027 ^c^	0.888 ± 0.048 ^c^	1.029 ± 0.015 ^b^	0.899 ± 0.021 ^c^	0.711 ± 0.019 ^d^
α (N·m/min)	−0.070 ± 0.007 ^b^	−0.099 ± 0.002 ^c^	−0.126 ± 0.011 ^d^	−0.100 ± 0.005 ^c^	−0.027 ± 0.006 ^a^	−0.031 ± 0.003 ^a^
β (N·m/min)	0.463 ± 0.039 ^b^	0.221 ± 0.030 ^e^	0.349 ± 0.014 ^cd^	0.303 ± 0.021 ^de^	0.613 ± 0.067 ^a^	0.403 ± 0.090 ^bc^
γ (N·m/min)	−0.020 ± 0.016 ^a^	−0.021 ± 0.006 ^a^	−0.025 ± 0.010 ^a^	−0.010 ± 0.007 ^a^	−0.023 ± 0.006 ^a^	0.007 ± 0.034 ^a^

Different superscript lowercase letters in the same row mean significantly differences (*p* < 0.05); TBF, TBCF and TBBF represent Tartary buckwheat flour, Tartary buckwheat core flour and Tartary buckwheat bran flour, respectively. W_bran_ is the weight proportion of TBBF in the blend four.

**Table 6:** Effect of W_bran_ on the color difference of steamed bread.

W_bran_ (%)	L*	a*	b*
0	81.88 ± 0.46 ^a^	−0.65 ± 0.02 ^f^	17.17 ± 0.20 ^c^
10	59.81 ± 0.16 ^b^	2.47 ± 0.07 ^e^	23.71 ± 0.33 ^a^
30	47.82 ± 0.48 ^c^	5.53 ± 0.04 ^a^	20.98 ± 0.23 ^b^
50	42.19 ± 0.23 ^d^	5.00 ± 0.08 ^b^	14.82 ± 0.18 ^d^
70	39.51 ± 0.22 ^e^	4.40 ± 0.03 ^c^	11.47 ± 0.16 ^e^
90	38.49 ± 0.11 ^f^	3.66 ± 0.13 ^d^	8.94 ± 0.09 ^f^

Different superscript lowercase letters in the same column mean significantly differences (*p* < 0.05); W_bran_ is the weight proportion of TBBF in the blend four.

**Table 7:** Effect of W_bran_ on parameters values of Mixolab curves of mixed dough.

W_bran_ (%)	Hardness	Springiness	Cohesiveness	Gumminess	Chewiness	Resilience
0	1651 ± 59 ^e^	0.945 ± 0.006 ^b^	0.846 ± 0.014 ^a^	1398 ± 41 ^e^	1321 ± 48 ^e^	0.558 ± 0.014 ^a^
10	1680 ± 42 ^e^	0.970 ± 0.026 ^a^	0.842 ± 0.014 ^a^	1415 ± 58 ^e^	1374 ± 92 ^e^	0.548 ± 0.014 ^a^
30	3554 ± 98 ^d^	0.904 ± 0.002 ^c^	0.808 ± 0.012 ^b^	2871 ± 43 ^d^	2596 ± 40 ^d^	0.485 ± 0.013 ^b^
50	8367 ± 378 ^c^	0.871 ± 0.008 ^d^	0.782 ± 0.002 ^c^	6542 ± 279 ^c^	5696 ± 290 ^c^	0.458 ± 0.003 ^c^
70	11116 ± 632 ^b^	0.872 ± 0.015 ^d^	0.783 ± 0.018 ^c^	8693 ± 294 ^b^	7577 ± 176 ^b^	0.448 ± 0.020 ^c^
90	19691 ± 752 ^a^	0.854 ± 0.008 ^d^	0.761 ± 0.018 ^c^	14192 ± 1441 ^a^	12116 ± 1228 ^a^	0.438 ± 0.017 ^c^

Different superscript lowercase letters in the same column mean significantly differences (*p* < 0.05); W_bran_ is the weight proportion of TBBF in the blend four.

**Table 8:** Effect of W_bran_ on parameters values of Mixolab curves of mixed dough.

**W_bran_ (%)**	**0**	**10**	**30**	**50**	**70**	**90**
Slice area	2819 ± 65 ^a^	2817 ± 40 ^a^	2258 ± 61 ^b^	1992 ± 13 ^c^	1918 ± 40 ^cd^	1842 ± 12 ^d^
Brightness	102.270 ± 7.079 ^a^	51.311 ± 0.884 ^b^	33.501 ± 3.704 ^c^	31.803 ± 1.568 ^c^	27.839 ± 0.484 ^c^	27.015 ± 1.515 ^c^
Cell contrast	0.796 ± 0.013 ^a^	0.622 ± 0.005 ^f^	0.649 ± 0.013 ^e^	0.688 ± 0.012 ^d^	0.722 ± 0.010 ^c^	0.766 ± 0.010 ^b^
Number of cells	1991 ± 148 ^a^	1558 ± 20 ^d^	1645 ± 26 ^cd^	1766 ± 88 ^bc^	1857 ± 71 ^ab^	1972 ± 41 ^a^
Area of cells	46.220 ± 0.978 ^a^	49.379 ± 0.150 ^b^	46.076 ± 1.039 ^b^	44.121 ± 0.439 ^c^	43.031 ± 0.343 ^c^	41.613 ± 0.334 ^d^
Wall thickness	0.447 ± 0.006 ^b^	0.485 ± 0.004 ^a^	0.450 ± 0.004 ^b^	0.420 ± 0.006 ^c^	0.406 ± 0.006 ^d^	0.395 ± 0.006 ^e^
Cell diameter	1.389 ± 0.050 ^b^	1.800 ± 0.043 ^a^	1.416 ± 0.057 ^b^	1.234 ± 0.077 ^c^	1.117 ± 0.036 ^d^	1.019 ± 0.025 ^e^
Cell volume	3.558 ± 0.205 ^cd^	6.984 ± 0.215 ^a^	5.164 ± 0.308 ^b^	4.034 ± 0.385 ^c^	3.378 ± 0.227 ^d^	2.747 ± 0.235 ^e^
Coarse cell volume	6.507 ± 1.157 ^b^	10.364 ± 0.493 ^a^	9.445 ± 0.626 ^a^	6.804 ± 0.752 ^b^	5.323 ± 0.147 ^c^	4.194 ± 0.302 ^c^
Cell elongation	1.618 ± 0.014 ^a^	1.532 ± 0.009 ^b^	1.556 ± 0.020 ^b^	1.564 ± 0.010 ^b^	1.607 ± 0.049 ^a^	1.643 ± 0.019 ^a^
Cell density	0.706 ± 0.046 ^d^	0.553 ± 0.010 ^e^	0.729 ± 0.014 ^d^	0.887 ± 0.048 ^c^	0.968 ± 0.018 ^b^	1.071 ± 0.029 ^a^

Different superscript lowercase letters in the same row mean significantly differences (*p* < 0.05); W_bran_ is the weight proportion of TBBF in the blend four.

## Data Availability

The data that support the findings of this study are available on request from the corresponding author. The data are not publicly available due to privacy or ethical restrictions.
